# Inhibitory effects of Mycoepoxydiene on macrophage foam cell formation and atherosclerosis in ApoE-deficient mice

**DOI:** 10.1186/s13578-015-0017-y

**Published:** 2015-05-26

**Authors:** Xiaochun Xia, Yang Li, Qiang Su, Zhengrong Huang, Yuemao Shen, Weihua Li, Chundong Yu

**Affiliations:** The First Affiliated Hospital of Xiamen University, Xiamen, Fujian 361005 China; State Key Laboratory of Cellular Stress Biology, Innovation Center for Cell Signaling Network, School of Life Sciences, Xiamen University, Xiang-An South Road, Xiamen, Fujian 360112 China; Medical College, Xiamen University, Xiamen, China; School of Pharmaceutical Sciences, Shandong University, Jinan, Shandong 250012 China

**Keywords:** Mycoepoxydiene, ox-LDL, Macrophage, Foam cell, Atherosclerosis

## Abstract

**Background:**

Mycoepoxydiene (MED) is a polyketide that can be isolated from a marine fungus and is associated with various activities, including antitumor and anti-inflammatory functions. However, its effects on atherosclerosis remain unknown. Macrophage-derived foam cells play crucial roles in the initiation and progression of atherosclerotic plaques. In this study, we investigated the effects of MED on oxidized low-density lipoprotein (ox-LDL)-induced macrophage foam cell formation and activation, and on high fat diet (HFD)-induced atherosclerosis in ApoE-deficient (*ApoE*^*−/−*^) mice.

**Results:**

Our findings show that MED could significantly inhibit ox-LDL-induced macrophage foam cell formation and suppress the expression of lectin-like oxidized low-density lipoprotein receptor-1 (LOX-1), which is a receptor for ox-LDL. Additionally, MED could significantly inhibit the secretion of proinflammatory cytokines, such as tumor necrosis factor (TNF-α), interleukin (IL)-6, and IL-1β. Mechanistically, MED inhibited NF-κB activation by blocking IκB-α degradation and reducing NF-κB DNA binding activity. Moreover, MED dramatically reduced the occurrence of HFD-induced atherosclerotic lesions in *ApoE*^*−/−*^ mice.

**Conclusions:**

Our study shows that MED can inhibit macrophage foam cell formation and activation by inhibiting NF-κB activation, thereby protecting *ApoE*^*−/−*^ mice from HFD-induced atherosclerosis. Our findings suggest that MED might be a potential lead compound for the development of antiatherosclerotic therapeutics.

**Electronic supplementary material:**

The online version of this article (doi:10.1186/s13578-015-0017-y) contains supplementary material, which is available to authorized users.

## Background

Atherosclerosis, a chronic inflammatory disease that is characterized by the accumulation of lipids and fibrous elements that result from interactions between vascular cells and inflammatory cells, is a leading cause of mortality in developed countries [1, 2]. Studies have shown that inflammation plays a critical role in all stages, from initiation through progression, and ultimately drives the thrombotic complications of atherosclerosis. Ox-LDL plays a critical role in triggering proinflammatory and pro-oxidant events in the initiation, propagation, and activation of atherosclerosis [3, 4]. Ox-LDL is taken up via the scavenger receptor expressed by macrophages, which turns macrophages into lipid-laden foam cells [5]. Additionally, ox-LDL stimulates macrophages to release proinflammatory cytokines such as IL-1β and TNF-α [6].

LOX-1, which is a lectin-like receptor for ox-LDL in endothelial cells, smooth muscle cells, and macrophages [7–9], plays important roles in atherosclerosis development [10]. The binding of ox-LDL to LOX-1 leads to ox-LDL uptake by macrophages and foam cell transformation [11]. NF-κB, which is a downstream factor of LOX-1, is a pivotal transcription factor involved in atherosclerosis. NF-κB activation has been observed at different stages of atherosclerosis, from plaque formation to its destabilization and rupture [12, 13]. LOX-1 can be dynamically up-regulated by various proinflammatory, pro-oxidative, and mechanical stimuli [14–16]. Many antiatherosclerotic drugs, such as stains, exert their function at least in part via direct or indirect down-regulation of LOX-1 expression [17, 18].

Mycoepoxydiene (MED) is a novel polyketide that can be isolated from the marine fungus *Diaporhte* sp. (*D*.sp.) HLY-1 and was discovered in submerged rotten leaves of *Kandelia candel* in a mangrove forest in Fujian Province, China [19]. It contains an α, β-unsaturated-lactone moiety and an oxygen-bridged cyclooctadiene core [20]. Previous studies have shown that MED could exert antimicrobial and antitumor activities [19, 21]. Recently, we have showed that MED could inhibit LPS-induced inflammatory responses, ovariectomy-induced osteoporosis, and allergic responses in mice [22–24]. In macrophages, MED can significantly inhibit the LPS-induced expression of proinflammatory mediators, such as TNF-α, IL-6, IL-1β and nitric oxide (NO) through blocking the activation of both NF-κB and MAPK pathways [22]. In mast cells, MED can significantly suppress antigen-stimulated degranulation and cytokine production by blocking the MAPK pathways. Because MED displays an anti-inflammatory activity, we wanted to determine whether it could inhibit atherosclerosis, which is related to the inflammatory response [25–27]. Herein, we show that MED can inhibit foam cell formation and cytokine production induced by ox-LDL in macrophages and protect *ApoE*^*−/−*^ mice from atherosclerosis by blocking the NF-κB signaling.

## Results

### MED significantly suppresses macrophage foam cell formation

**Fig. 1:**
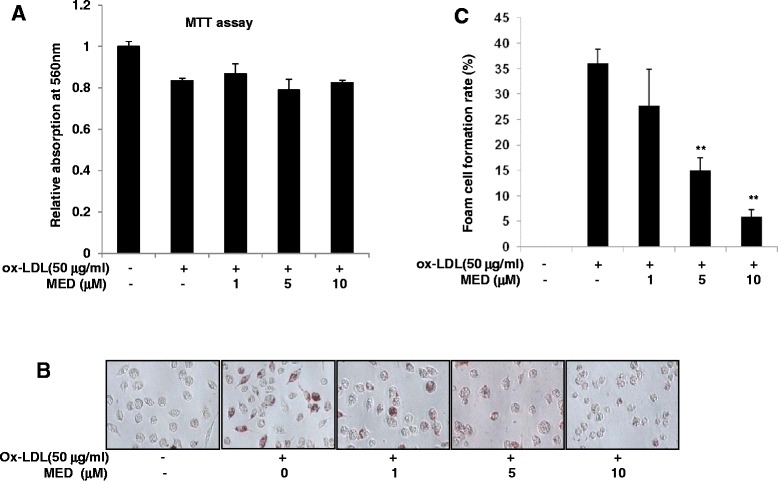
MED inhibits ox-LDL-induced foam cell formation. RAW264.7 cells were treated with various doses of MED along with ox-LDL (50 μg/ml) for 24 h, and the MTT assay and oil red staining experiments were performed. **a** MED showed no cytotoxicity to RAW264.7 cells. Cell viability was measured by the MTT assay. **b** and **c** MED inhibited ox-LDL-induced foam cell formation. **b**, Representative images are shown. **c**, Oil red-positive RAW264.7 cells were quantified. Values are expressed as the means ± SD from three independent experiments; **p < 0.01

Foam cells are pathogenic cells in atherosclerosis that are derived from monocytes/macrophages and vascular smooth muscle cells. RAW264.7 cells, which share many features in common with macrophages, can be induced to form foam cells by ox-LDL *in vitro*. To explore whether MED affected foam cell formation, RAW264.7 cells were treated with ox-LDL in the absence or presence of different concentrations of MED for 24 h and were then stained with Oil-red O solution. First, we detected the cytotoxicity of MED to RAW 264.7 cells using a MTT assay. As shown in Fig. 1a, MED showed no cytotoxicity to RAW 264.7 cells in a concentration range between 1 and 10 μM; therefore, these concentrations of MED were used in the subsequent *in vitro* experiments. A total of 35 % of RAW264.7 cells displayed foamy characteristics with oil red staining of lipid droplets after incubation with ox-LDL for 24 h, but MED treatment significantly reduced the foam cell formation in a dose-dependent manner (Fig. 1b, c).

### MED inhibits ox-LDL-induced LOX-1 expression in macrophages

**Fig. 2:**
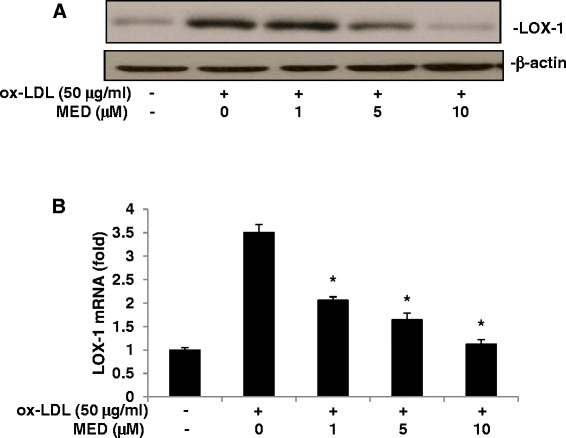
MED attenuates ox-LDL-induced LOX-1 expression. RAW264.7 cells were treated with ox-LDL (50 μg/ml) in the absence or presence of MED for 24 h. **a** LOX-1 protein levels were measured by western blot analysis. **b** LOX-1 mRNA levels was evaluated by real-time PCR. Values indicate means ± SD from three independent experiments; *p < 0.05

LOX-1 is a lectin-like receptor for ox-LDL in endothelial cells, smooth muscle cells, and macrophages, which are three important cell types involved in the development of atherosclerosis [10]. LOX-1 expression can be induced by ox-LDL in a feed-forward fashion. To determine whether MED can inhibit foam cell formation by blocking ox-LDL-induced LOX-1 expression, RAW 264.7 cells were treated with ox-LDL in the absence or presence of MED for 24 h; the LOX-1 protein levels were then evaluated by western blotting. As shown in Fig. 2a, MED significantly inhibited the LOX-1 protein levels in a dose-dependent manner. To determine whether the reduced expression level of LOX-1 could be attributed to the reduced mRNA levels of LOX-1, real-time PCR was performed. As shown in Fig. 2b, MED significantly suppressed the mRNA levels of LOX-1, which indicated that MED may inhibit ox-LDL-induced LOX-1 expression at the transcriptional level.

### MED can suppress proinflammatory cytokine expression in macrophages induced by ox-LDL

**Fig. 3:**
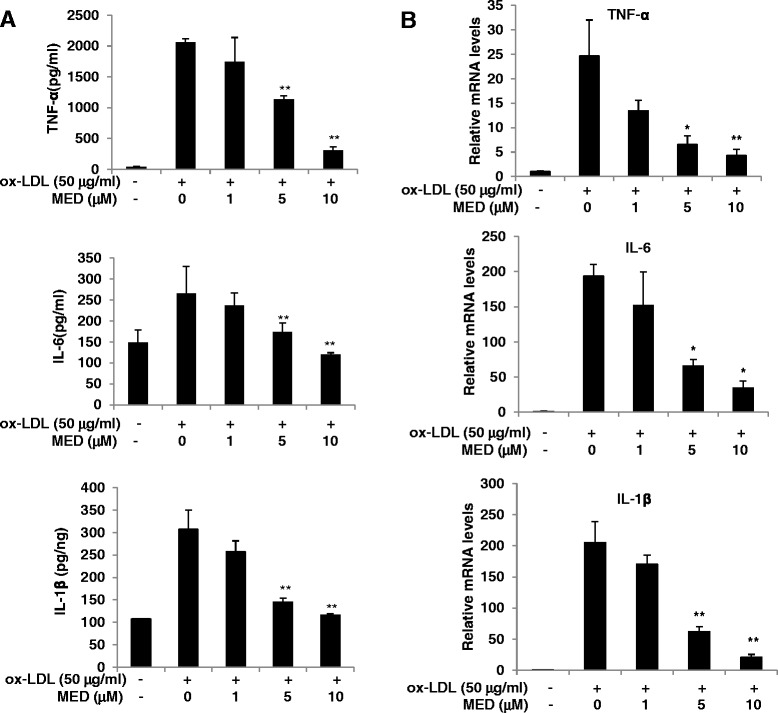
MED significantly inhibits ox-LDL-induced production of TNF-α, IL-6, and IL-1β. **a** RAW264.7 cells were stimulated with ox-LDL in the presence of different concentrations of MED for 24 h. The amounts of TNF-α, IL-6, and IL-1β were measured using ELISA assay kits. **b** The mRNA levels of TNF-α, IL-6, and IL-1β were measured by real-time PCR. Values indicate means ± SD from three independent experiments. **p < 0.01, *p < 0.05

It has been demonstrated that macrophages are abundant in plaque lesions and play a central role in atherosclerosis development [28]. In addition to inducing foam cell formation, ox-LDL can activate macrophages to produce proinflammatory cytokines, such as TNF-α, IL-6, and IL-1β, to promote atherosclerosis. To examine whether MED could affect the ox-LDL-induced production of TNF-α, IL-6, and IL-1β in macrophages, the production of these cytokines by ox-LDL-stimulated macrophages was measured in the absence or presence of MED. As shown in Fig. 3a, MED significantly inhibited the ox-LDL-induced production of TNF-α, IL-6, and IL-1β. Furthermore, we found that MED significantly suppressed the mRNA levels of TNF-α, IL-6, and IL-1β in RAW 264.7 cells (Fig. 3b), which indicated that MED might inhibit ox-LDL-induced proinflammatory cytokine expression at the transcriptional level.

### MED inhibits ox-LDL induced activation of NF-κB signaling in macrophages

**Fig. 4:**
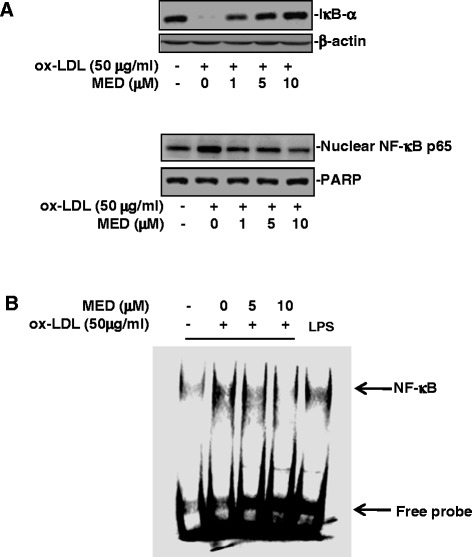
MED inhibits ox-LDL-induced activation of NF-κB signaling. **a** and **b** RAW264.7 cells were stimulated with ox-LDL in the absence or presence of MED for 3 h, and then western blot analysis of IκB-α and nuclear p65 was performed. Representative images of three independent experiments are shown. **a**, The effect of MED on ox-LDL-induced IκB-α degradation and nuclear NF-κB p65 expression. **b**, The effect of MED on ox-LDL-induced NF-κB DNA binding activity. RAW264.7 cells were stimulated with ox-LDL in the absence or presence of MED for 3 h, and then nuclear extracts were obtained for EMSA

It has been shown that ox-LDL can induce the expression of LOX-1 and proinflammatory cytokines, such as IL-1β and TNF-α, through the activation of NF-κB signaling [2, 29]. To explore whether MED could inhibit NF-κB signaling, the effects of MED on ox-LDL-induced NF-κB activation in RAW 264.7 cells were examined. As shown in Fig. 4a and b, MED significantly suppressed ox-LDL-induced IκB-α degradation, nuclear p65 expression, and the DNA binding activity of NF-κB at concentrations of 5 and 10 μM in macrophages, which indicated that MED could inhibit the activation of NF-κB signaling.

### MED effectively reduces atherosclerotic lesions in ApoE^−/−^ mice

**Fig. 5:**
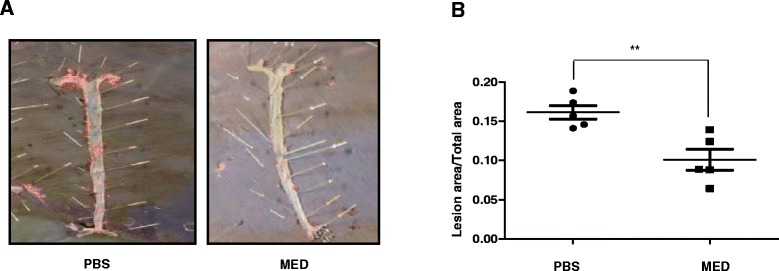
MED effectively reduces atherosclerotic lesions in HFD-fed *ApoE* ^*−/−*^ mice. **a** HFD-fed mice were injected with PBS or 4 mg/kg/day MED every other day for 8 weeks, respectively. At the end points, mice were sacrificed, and atherosclerotic lesions in the descending aorta were detected by Sudan IV staining. **b** Quantitative analysis of atherosclerotic lesion sizes. Data are presented as the means ± S.D;*n* = 5 per group. **p < 0.01

Because high-fat diet (HFD)-induced arterial lesions in *ApoE*^*−/−*^ mice have many features in common with human atherosclerosis, *ApoE*^*−/−*^ mice are considered to be a good animal model of atherosclerosis [30]. Because MED could effectively inhibit ox-LDL-induced macrophage foam cell formation and activation *in vitro*, we assessed whether MED could suppress HFD-induced atherosclerosis in *ApoE*^*−/−*^ mice. *ApoE*^*−/−*^ mice were fed with a high-fat diet, and vehicle (PBS) or MED (4 mg/kg/day) was injected every 2 days for 2 months, then, the atherosclerotic lesions in the descending aorta were detected by Sudan IV staining. As shown in Fig. 5a and b, MED significantly suppressed the area of lesions compared with the PBS control group, which demonstrated that MED can suppress atherosclerosis *in vivo*. Additionally, we found that there was no significant difference in body weight gain between the PBS control group and MED group during the 8 weeks of HFD feeding (Additional file 1: Figure S1), which indicated that MED caused no apparent toxicity in mice after long-term MED treatment.

**Fig. 6:**
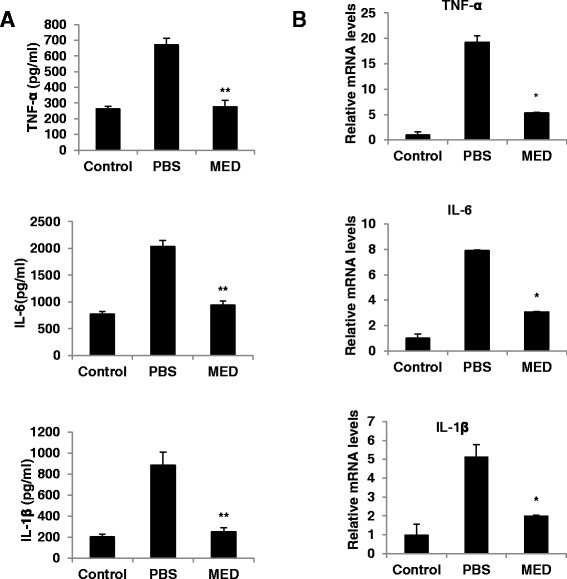
Inhibitory effects of MED on pro-inflammatory cytokine expression in mice. **a** The amounts of TNF-α, IL-6, and IL-1β in plasma were measured using ELISA assay kits. **b** Expression levels of the inflammatory genes, including TNF-α, IL-6, and IL-1β, were measured by real-time PCR in the aortic arch isolated from mice; Control, normal chow-fed group; PBS, HFD-fed and PBS-treated group; MED, HFD-fed and MED treated group. Data indicate means ± S.D; *n* = 5 per group; **p < 0.01, *p < 0.05

To determine whether MED could suppress inflammation in mice after 8 weeks of HFD feeding, the levels of inflammatory cytokines, including TNF-α, IL-6, and IL-1β, in plasma and the aortic arch of HFD-fed ApoE-deficient mice were measured. As shown in Fig. 6a and b, MED suppressed the protein levels of TNF-α, IL-6, and IL-1β in plasma and the mRNA levels of TNF-α, IL-6, and IL-1β in the aortic arch. Furthermore, MED suppressed nuclear p65 expression in the aortic arch of HFD-fed ApoE-deficient mice (Additional file 2: Figure 2S). These results indicate that MED can inhibit atherosclerosis at least in part by suppressing inflammation *in vivo*.

## Discussion

Atherosclerosis is considered a type of chronic inflammation. The differentiation of macrophages to lipid-laden foam cells is a pivotal process in the development of the atherosclerosis [31]. The transformation of foam cells can be attributed to the dysregulated uptake of modified LDL by macrophages [32, 33]. Ox-LDL is a critical factor in the initiation and progression of atherosclerosis, especially for foam cell formation. It is thought that uptake of ox-LDL by macrophage scavenger receptors is largely responsible for disease progression. As a receptor for ox-LDL, LOX-1 accumulates in human and animal atherosclerotic lesions *in vivo* [34]. It has been shown that LOX-1 functions as a proinflammatory adhesion molecule in leukocytes and platelets [35]. LOX-1 affects several inflammatory stages of atherosclerosis, such as macrophage foam cell formation and activation [16]. It has been reported that the deletion of LOX-1 reduces atherogenesis in LDLR-knockout mice that are fed a high cholesterol diet [36], thus highlighting the important role of LOX-1 in atherosclerosis. In our present study, MED could significantly inhibit RAW 264.7 cells transformation into foam cells and LOX-1 expression induced by ox-LDL, which indicated that the suppression of LOX-1 expression by MED contributes to its inhibitory effects on macrophage foam cell formation.

It has been shown that LOX-1 is involved in inflammatory reactions and is an important mediator of inflammation [37]. Macrophages produce abundant amounts of pro-inflammatory cytokines, such as TNF-α, IL-6, and IL-1β, to promote atherosclerosis. Herein, we have shown that MED can significantly inhibit the ox-LDL-induced expression of TNF-α, IL-6, and IL-1β both in RAW264.7 cells and in plaque lesion areas, which suggests that MED can suppress inflammatory responses in atherosclerosis.

NF-κB is a transcription factor involved in several inflammatory diseases, including atherosclerosis. The activation of NF-κB has been observed at different stages of atherosclerosis [13]. The binding of ox-LDL to LOX-1 can activate NF-κB signaling, thus leading to enhanced macrophage foam cell formation and proinflammatory cytokine production. Our data show that MED could significantly inhibit ox-LDL/LOX-1-induced activation of NF-κB signaling, which suggests that MED inhibits macrophage foam cell formation and activation at least in part by blocking NF-κB signaling.

The most significant finding of this current study is that MED could dramatically suppress atherosclerosis in *ApoE*^*−/−*^ mice fed a high fat diet. In addition to inhibiting inflammation and foam cell formation, we found that MED could significantly reduce the levels of blood fats, such as total triglyceride, total cholesterol, high-density lipoprotein, and low-density lipoprotein in mice (Additional file 3: Figure S3). These features might all contribute to the anti-atherogenic activity of MED. Therefore, MED exhibits the anti-atherogenic activity both *in vitro* and *in vivo*, which suggests that MED might represent a novel drug candidate for the effective treatment of atherosclerosis.

Collectively, our results demonstrate that MED, a novel marine microbial compound, can inhibit macrophage foam cell formation and activation *in vitro* and suppress atherosclerosis in *ApoE*^*−/−*^ mice, at least in part by inhibiting NF-κB activation. Our findings suggest that MED represents is a potential lead compound for the development of novel anti-atherosclerotic drugs.

## Materials and methods

### Materials

MED was isolated from the fermentation broth of D. sp. HLY-1, as previously described [19]. The identity of MED was confirmed by HRMS and ^1^H and ^13^C NMR analysis, and the purity of MED exceeded 95.7 % according to HPLC analysis. MED was dissolved in dimethylsulfoxide (DMSO) and stored at −20 °C. DMSO, MTT, oil red O, and anti-β-actin antibody were obtained from Sigma-Aldrich (Sigma, St. Louis, MO, USA); IL-1β ELISA kit, IL-6 ELISA kit, and TNF-α ELISA kit were obtained from eBioscience (eBioscience, SanDiego, CA, USA); DMEM was purchased from Gibco (Gibco, Grand Island,NY, USA); fetal bovine serum (FBS) was obtained from Hyclone (Thermo Scientific, IL, USA); Ox-LDL was purchased from Unionbiol (Beijing, China); anti-IκB-α antibody was obtained from Santa Cruz Biotechnology (Santa Cruz, CA, USA); antibodies against p65 and PARP were purchased from Cell Signaling Technology (Danvers, MA, USA); antibody against LOX-1 was obtained from R&D systems (Minneapolis, MN, USA); high-fat diet was produced by Vital River Laboratories (Beijing, China) according to the formula from Harlan (TD.88137).

## Methods

### Cell culture

Murine macrophage cell RAW 264.7 cells were purchased from the American Type Cell Culture Collection. Cells were cultured in DMEM supplemented with 10 % fetal bovine serum (FBS).

### Cytotoxicity assay

The cytotoxicity of MED was analyzed using a MTT assay. A total of 7 × 10^3^ cells/well were seeded into a 96-well plate. After incubation with MED for 24 h, 10 μl MTT (5 mg/ml, Sigma) was added to each well. Plates were incubated for 4 h before adding of 100 μl lysis buffer (10 % SDS in 0.01 M HCl). Absorbance was measured at 560 nm using a microplate reader.

### Western blot analysis

Lysates for western blot analysis were prepared from cells and aortic tissues. A total of 30 μg protein lysates of each sample were subjected to SDS-PAGE and transferred onto nitrocellulose membranes. Blots were incubated with appropriate specific primary antibodies overnight at 4 °C. After washing three times for 15 min each with TBST (TBS + 0.1 % Tween20), blots were incubated with horseradish-peroxidase-conjugated secondary antibody (Pierce, Rockford, IL, USA) and visualized by chemiluminescence. Band densities were quantified by densitometry using the Scion Image software and were normalized to the β-actin and PARP levels.

### Quantitative real-time PCR

Total RNA was isolated using Trizol reagent (Invitrogen) according to the manufacturer’s instructions. The cDNA was synthesized from 2 μg total RNA using MMLV transcriptase (ToYoBo, Shanghai, China) with random primers, real-time PCR was performed using SYBR Premix ExTaq (TaKaRa, Dalian, China). Quantification was normalized to the amount of endogenous GAPDH.

### Cytokine assays

Cells were seeded in 96-well plates 24 h prior to ox-LDL and MED treatment, and the concentrations of TNF-α, IL-6, and IL-1β were determined using ELISA kits (eBioscience, San Diego, CA) according to the manufacturer’s instructions.

### High-density lipoprotein (HDL), low-density lipoprotein (LDL), triglyceride (TG), and total cholesterol (TC)

Mice were euthanized and blood was collected. HDL, LDL, TG, and TC were measured using ELISA kits according to the manufacturer’s instructions.

### Nuclear protein extraction

After ox-LDL and MED treatments, cells were collected and then Buffer A (10 mM HEPES (pH 7.9), 10 mM KCl, 0.1 mM EDTA, 1 mM DTT, and 0.5 mM PMSF) was added. After incubation on ice for 20 min, cells were suspended in Buffer A with 2.5 % NP- 40, vortexed for 10 s and then centrifuged at 12,000 × *g* for 5 min at 4 °C. Supernatants were collected to detect cytoplasmic proteins. Buffer B (20 mM HEPES (pH 7.9), 0.4 M NaCl, 1 mM EDTA, 1 mM DTT, and 1 mM PMSF) was used to resuspend the precipitates, and the suspension was vortexed for 25 min at 4 °C and then centrifuged at 12,000 × *g* for 5 min at 4 °C. Supernatants were collected as nuclear extracts, and the protein concentrations were measured using the Bradford protein assay (Bio-Rad, Hercules, CA).

### Electrophoretic mobility shift assay

A total of 10 μg nuclear extract was incubated with 20 nM biotin-labeled double- stranded oligonucleotide probes for 20 min at room temperature, and then were separated on a non-denaturing 6 % (w/v) polyacrylamide gel. The biotin-labeled oligonucleotide probes (NF-κB: 5′ -AGT TGA GGG GAC TTT CCC AGG C-3′, 3′ -TCA ACT CCC CTG AAA GGG TCC G-5′) were transferred from the polyacrylamide gel onto a nylon membrane. Bands were detected with a LightShift chemiluminescent EMSA kit (Pierce, Rockford, IL) according to the manufacturer’s instruction.

### High-fat diet-induced atherosclerosis in ApoE^−/−^ mice

Eight-week-old *ApoE*^*−/−*^ mice were fed a high-fat diet for 12 weeks to induce atherosclerosis. All experiments were approved by the Animal Care and Use Committee of Xiamen University (Protocol Number: XMULAC 20120001). Every effort was made to reduce animal suffering.

### Atherosclerotic lesion analysis

Mice were euthanized and their hearts and aortas were isolated. The degree of atherosclerosis was assessed by determining lesion sizes on both pinned open aortas and serial cross-sections through the aortic root as previously described but with some modification [38–40]. The aorta was opened longitudinally along the ventral midline from the iliac arteries to the aortic root. After the branching vessels were treated, the aorta was pinned out flat on a black wax surface. Lesions were treated with 70 % ethanol and then stained with Sudan IV for 15 min, destained with 80 % ethanol, and then washed with PBS. Aortic images were analyzed using the Adobe Photoshop software; data are reported as the percentage of the aortic surface covered by lesions.

### Statistical analysis

Data were collected from several independent experiments, with three replicates per experiment. All data were analyzed by one-way Anova with post-Tukey’s post-test with the SPSS 16.0 software package: *p* < 0.05 and *p* < 0.01 were considered to indicate statistically significant differences. Bars in the graph represent standard deviation (S.D.).

## Additional files

Additional file 1: Figure S1. There is no significant difference in body weight change between PBS control group and MED group during 8 weeks of HFD feeding. Additional file 2: Figure S2. Inhibitory effects of MED on NF-κB activation in aortic arch tissue isolated from mice. Nuclear proteins were extracted from aortic arch tissue. Nuclear NF-κB p65 was detected by Western blot. Representative images are shown from three independent experiments. (Control: normal chow-fed group, PBS: HFD-fed and PBS treated group, MED: HFD-fed and MED treated group). Additional file 3: Figure S3. MED decreases the levels of blood fats in mice. Mice were euthanized and blood was collected. The levels of total triglyceride, total cholesterol, high density lipoprotein, and low density lipoprotein were measured using ELISA kits. (Control: normal chow-fed group, PBS: HFD-fed and PBS treated group, MED: HFD-fed and MED treated group). Values are shown as the mean ± SD from three independent experiments. **p* < 0.05vs PBS group.
