# Supplementary material for: Inhibitory effects of Mycoepoxydiene on macrophage foam cell formation and atherosclerosis in ApoE-deficient mice
Source: Cell Biosci. 2015 May 26;5:23. doi: 10.1186/s13578-015-0017-y (PMC4455339; doi:10.1186/s13578-015-0017-y)
Supplement: Additional file 1: Figure S1. — There is no significant difference in body weight change between PBS control group and MED group during 8 weeks of HFD feeding. [file 13578_2015_17_MOESM1_ESM.pptx]

## Slide 1
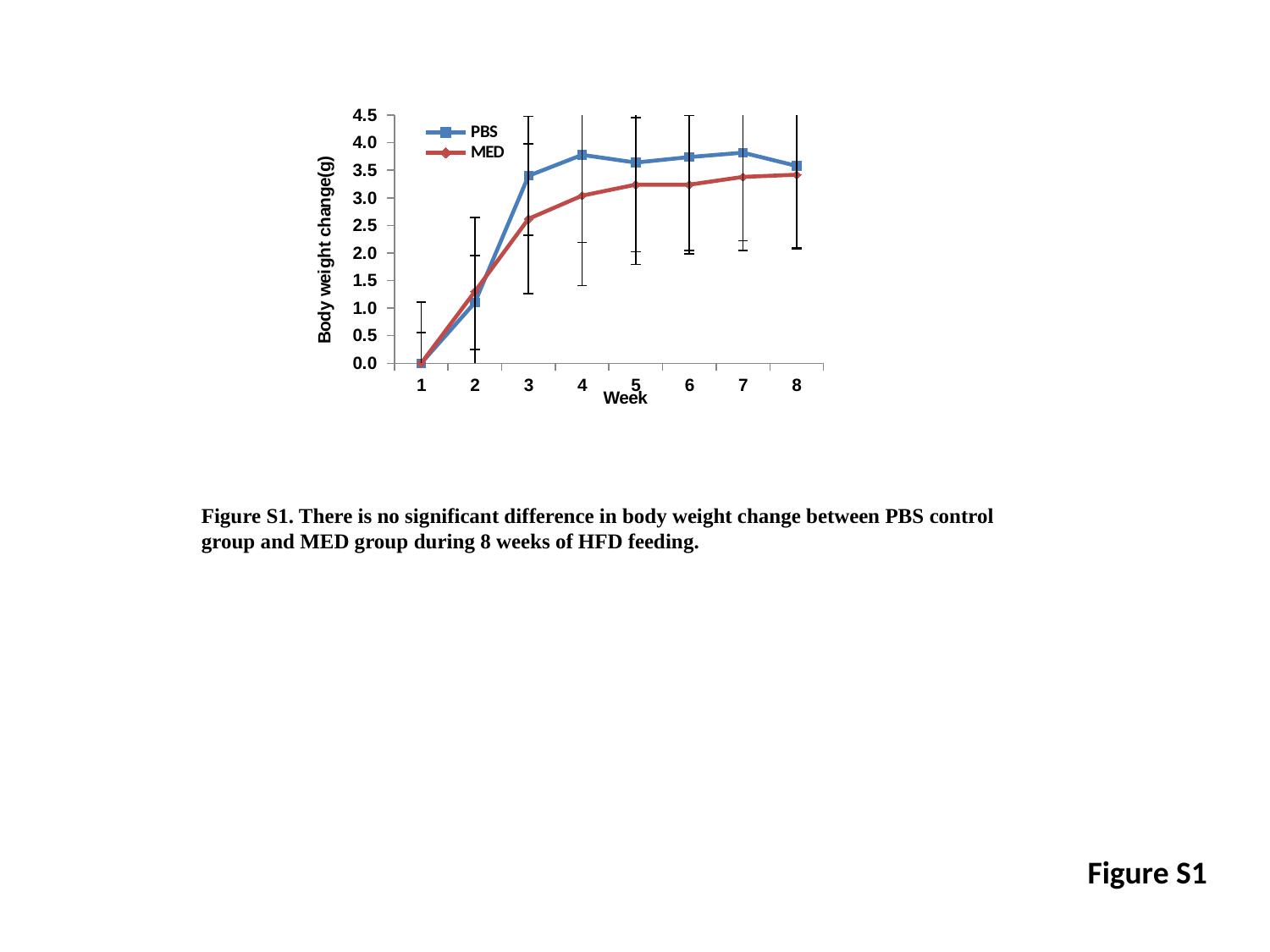

### Chart
| Category | PBS | MED |
|---|---|---|Figure S1. There is no significant difference in body weight change between PBS control group and MED group during 8 weeks of HFD feeding.
Figure S1
