# Supplementary material for: Inhibitory effects of Mycoepoxydiene on macrophage foam cell formation and atherosclerosis in ApoE-deficient mice
Source: Cell Biosci. 2015 May 26;5:23. doi: 10.1186/s13578-015-0017-y (PMC4455339; doi:10.1186/s13578-015-0017-y)
Supplement: Additional file 2: Figure S2. — Inhibitory effects of MED on NF-κB activation in aortic arch tissue isolated from mice. Nuclear proteins were extracted from aortic arch tissue. Nuclear NF-κB p65 was detected by Western blot. Representative images are shown from three independent experiments. (Control: normal chow-fed group, PBS: HFD-fed and PBS treated group, MED: HFD-fed and MED treated group). [file 13578_2015_17_MOESM2_ESM.pptx]

## Slide 1
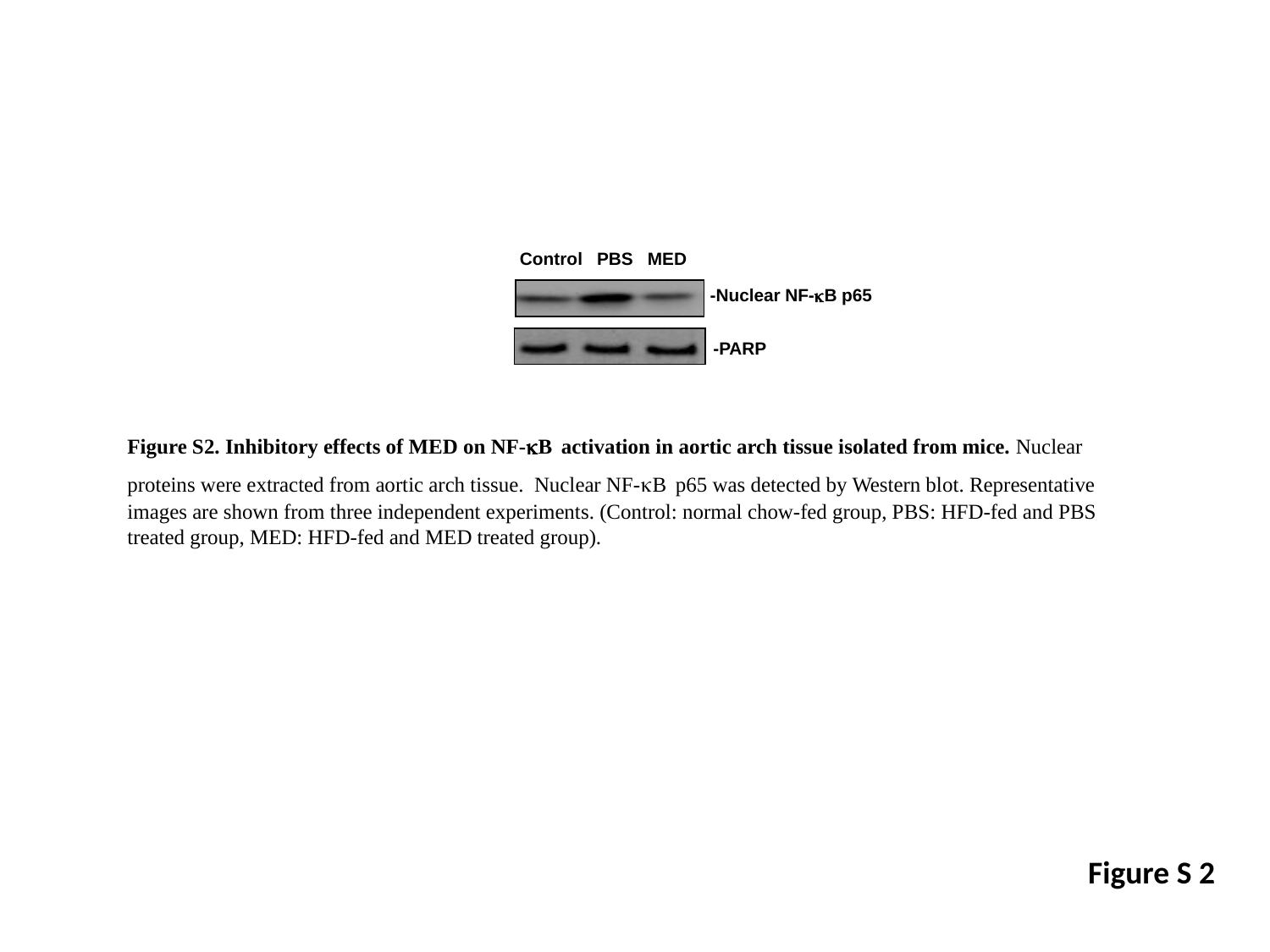

Control PBS MED
-Nuclear NF-κB p65
-PARP
Figure S2. Inhibitory effects of MED on NF-κB activation in aortic arch tissue isolated from mice. Nuclear proteins were extracted from aortic arch tissue. Nuclear NF-κB p65 was detected by Western blot. Representative images are shown from three independent experiments. (Control: normal chow-fed group, PBS: HFD-fed and PBS treated group, MED: HFD-fed and MED treated group).
Figure S 2
