# Supplementary material for: Inhibitory effects of Mycoepoxydiene on macrophage foam cell formation and atherosclerosis in ApoE-deficient mice
Source: Cell Biosci. 2015 May 26;5:23. doi: 10.1186/s13578-015-0017-y (PMC4455339; doi:10.1186/s13578-015-0017-y)
Supplement: Additional file 3: Figure S3. — MED decreases the levels of blood fats in mice. Mice were euthanized and blood was collected. The levels of total triglyceride, total cholesterol, high density lipoprotein, and low density lipoprotein were measured using ELISA kits. (Control: normal chow-fed group, PBS: HFD-fed and PBS treated group, MED: HFD-fed and MED treated group). Values are shown as the mean ± SD from three independent experiments. *p < 0.05vs PBS group. [file 13578_2015_17_MOESM3_ESM.pptx]

## Slide 1
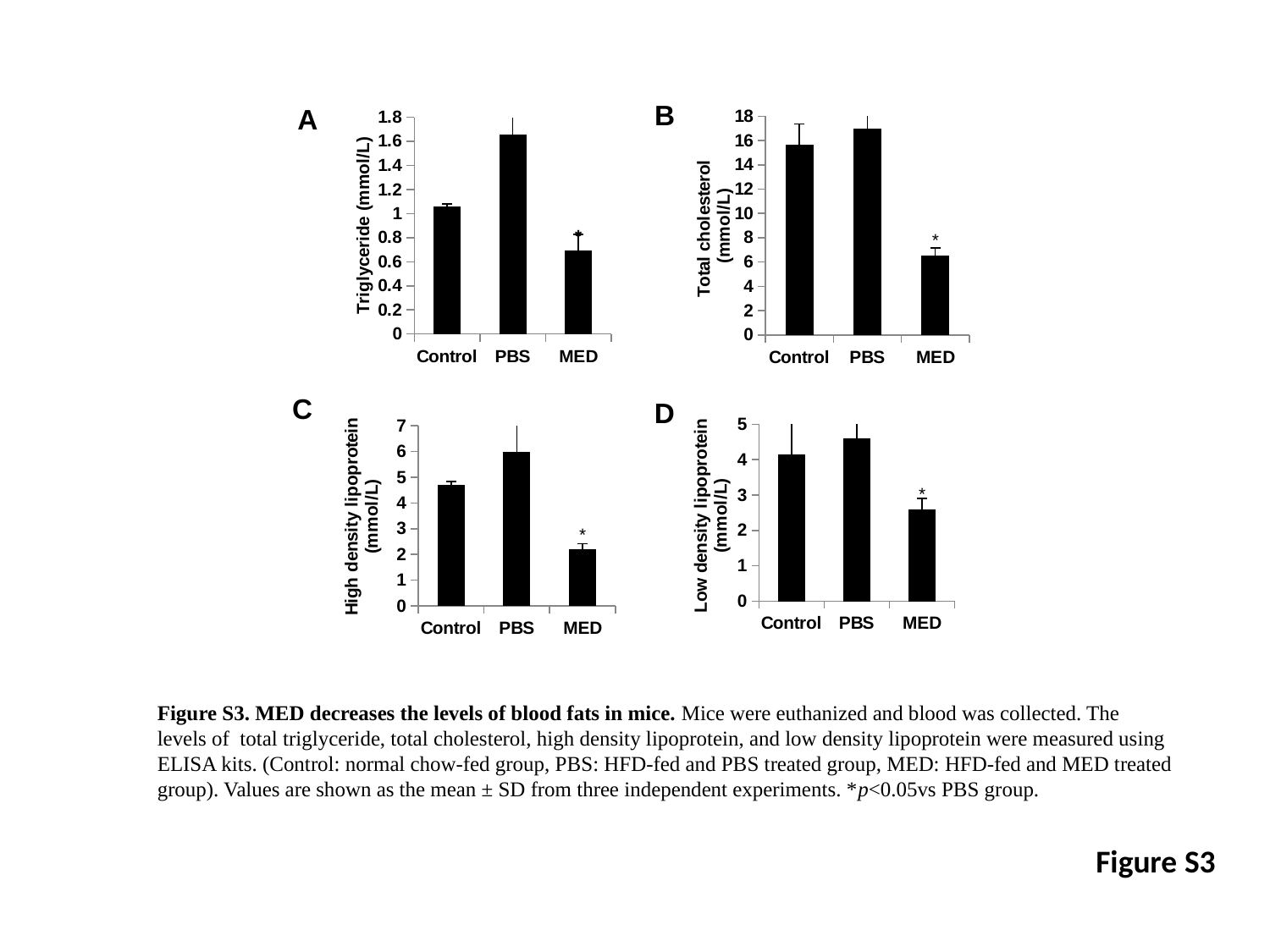

B
A
### Chart
| Category | |
|---|---|
| Control | 1.0521427094105509 |
| PBS | 1.65328335056877 |
| MED | 0.688975222993064 |
### Chart
| Category | |
|---|---|
| Control | 15.6286 |
| PBS | 16.978 |
| MED | 6.506548148148148 |C
D
### Chart
| Category | |
|---|---|
| Control | 4.129252173913049 |
| PBS | 4.597704347826071 |
| MED | 2.5860041407867493 |
### Chart
| Category | |
|---|---|
| Control | 4.682297496318117 |
| PBS | 5.978085419734904 |
| MED | 2.1716421895861067 |Figure S3. MED decreases the levels of blood fats in mice. Mice were euthanized and blood was collected. The levels of total triglyceride, total cholesterol, high density lipoprotein, and low density lipoprotein were measured using ELISA kits. (Control: normal chow-fed group, PBS: HFD-fed and PBS treated group, MED: HFD-fed and MED treated group). Values are shown as the mean ± SD from three independent experiments. *p<0.05vs PBS group.
Figure S3
